# An Experimental and Clinical Physiological Signal Dataset for Automated Pain Recognition

**DOI:** 10.1038/s41597-024-03878-w

**Published:** 2024-09-27

**Authors:** Philip Gouverneur, Aleksandra Badura, Frédéric Li, Maria Bieńkowska, Luisa Luebke, Wacław M. Adamczyk, Tibor M. Szikszay, Andrzej Myśliwiec, Kerstin Luedtke, Marcin Grzegorzek, Ewa Piętka

**Affiliations:** 1 Institute of Medical Informatics, University of Lübeck, Ratzeburger Allee 160, 23562 Lübeck, Germany; 2 Faculty of Biomedical Engineering, Silesian University of Technology, Roosevelta 40, 41-800 Zabrze, Poland; 3 Institute of Health Sciences, Department of Physiotherapy, Pain and Exercise Research Luebeck (P.E.R.L.), University of Lübeck, Ratzeburger Allee 160, 23562 Lübeck, Germany; 4 Center of Brain, Behavior and Metabolism (CBBM), University of Lübeck, Ratzeburger Allee 160, 23562 Lübeck, Germany; 5 Laboratory of Pain Research, Institute of Physiotherapy and Health Sciences, Academy of Physical Education in Katowice, Mikołowska 72a, 40-065 Katowice, Poland; 6 Division of Behavioral Medicine and Clinical Psychology, Cincinnati Children’s Hospital Medical Center, Cincinnati, OH US; 7 Laboratory of Physiotherapy and Physioprevention, Institute of Physiotherapy and Health Sciences, Academy of Physical Education in Katowice, Mikołowska 72a, 40-065 Katowice, Poland; 8 German Research Center for Artificial Intelligence (DFKI), Ratzeburger Allee 160, 23562 Lübeck, Germany

**Keywords:** Health care, Machine learning

## Abstract

Access to large amounts of data is essential for successful machine learning research. However, there is insufficient data for many applications, as data collection is often challenging and time-consuming. The same applies to automated pain recognition, where algorithms aim to learn associations between a level of pain and behavioural or physiological responses. Although machine learning models have shown promise in improving the current gold standard of pain monitoring (self-reports) only a handful of datasets are freely accessible to researchers. This paper presents the PainMonit Dataset for automated pain detection using physiological data. The dataset consists of two parts, as pain can be perceived differently depending on its underlying cause. (1) Pain was triggered by heat stimuli in an experimental study during which nine physiological sensor modalities (BVP, 2×EDA, skin temperature, ECG, EMG, IBI, HR, respiration) were recorded from 55 healthy subjects. (2) Eight modalities (2×BVP, 2×EDA, EMG, skin temperature, respiration, grip) were recorded from 49 participants to assess their pain during a physiotherapy session.

## Background & Summary

Although being of utmost importance for complete anamnesis, successful treatment, and accurate pain management^1^, the assessment of pain in the clinical context has remained unchanged for the last decades. The current gold standard of estimating a level of pain in subjects, that is even considered to be the fifth vital sign^2,3^, is given by self-reports^4^. Here, patients are asked to communicate their subjective pain impression. To enable comparability, several variations of the so-called pain rating scales have been introduced in the past. Their purpose is to provide a fixed setup to reliably express pain based on given anchors or a framework, for example, in the form of Numerical Rating Scales (NRSs) by translating pain intensity into a single number, or like in case of Visual Analogue Scale (VAS), into a point on a line with fixed intervals. Despite being a golden standard, the scale-based pain assessment may be a challenge for clinical applications^4^, for patients with cognitive impairment, young children, elderly people, or others for whom verbal communication is difficult. Considering the multidimensionality of the phenomenon, it is impossible to objectively measure the level of pain using solely VAS, NRS, or other scale because it is burdened with personal factors. Moreover, their usefulness for continuous pain monitoring is limited. This is particularly true when pain needs to be monitored over a long period of time, where frequent, continuous recording of pain intensity is almost impossible for healthcare professionals. Thus, automatic systems for pain recognition may become an essential support in medical procedures, especially for demanding patients.

Similar to various other areas of medicine, there is hope in automating tasks that are currently performed manually (such as the one of pain assessment) by leveraging machine learning algorithms to learn associations between a level of pain and physiological responses. To do this, a curated and diverse dataset is a fundamental requirement for conducting machine learning studies and understanding associations between pain and specific responses. The successful acquisition of a pain dataset involves the induction of pain in either an experimental or naturalistic setting, with a strategy to quantify the received pain level. Previous work has shown that biomarkers in the form of some specific physiological responses such as Electrodermal Activity (EDA) or Electromyogram (EMG) can be a good indicator of painful sensations in humans^5–7^. Unfortunately, responses to pain have often only been recorded in relation to an objective pain level. Examples include the Biopotential and Video (BioVid) Heat Pain Database (BVDB)^8^ which solely uses the stimulus intensity as a label, the *SenseEmotion*^9^ dataset relying just on heat stimuli, or the McMaster University and University of Northern British Columbia (UNBC)-McMaster shoulder pain expression archive dataset^10^ that uses a complex setup with intrusive data collection. In addition, there are several public datasets on experimentally induced pain, but none based on clinical procedures. Although the UNBC-McMaster dataset and EmoPain Dataset (EmoPain)^11^ include data from physiotherapy patients with chronic back pain, they are based on fixed range-of-motion tests performed by the patients themselves. The studies were carried out in a laboratory environment with a designed scenario, and they do not represent a naturalistic setup.

This work therefore presents the *PainMonit Dataset* for automated pain detection using physiological sensor data. It is made publicly available for researchers to further investigate the relationship between pain and physiological sensor modalities. As pain is a complex phenomenon^12^ that can have different dimensions and magnitudes depending on its underlying cause, and may evoke different responses due to varying qualities^6^, the presented dataset consists of time series data recorded by wearable devices from healthy subjects under two types of pain. On the one hand, pain was artificially induced by heat stimulation during an experimental study protocol. On the other hand, physiological responses to pain were also recorded during a physiotherapy session to provide data in a naturalistic setup. This proposed multi-context dataset gives the opportunity to experiment with knowledge transfer between an experimental and clinical setup using for instance transfer learning that aims at extracting and transferring knowledge from a source to a target domains^13^, or multitask learning that can obtain joint feature representations for the clinical and experimental setup by training models to solve both pain recognition tasks on both datasets^14^. This could enable the verification of how experimental and clinical pain differ, and how machine learning models trained in laboratory-based conditions generalise to a naturalistic setup.

The *PainMonit Dataset* provides additional benefits compared to other datasets for automated pain recognition^15^: (1) The dataset comprises the data from 104 subjects in total, each of which comes with multiple pain stimulations at various levels. The only dataset with more subjects than ours is the Experimentally Induced Thermal and Electrical (X-ITE) dataset^6^ with 134 subjects, which is not yet available to the public. (2) The clinical part of the PainMonit dataset provides recordings obtained in a real clinical context. To our best knowledge, it is the first publicly available dataset collected using real medical procedures. (3) It contains two different pain elicitation methods (heat and touch/pressure). Contrary to X-ITE, that also used two stimuli (heat and electricity), one of our pain induction methods was not experimental. Multi-context pain data analysis may lead to answering the essential questions that have not been addressed yet: how does experimentally induced pain differ from real-context pain, and is it possible to develop more robust machine learning models using both parts. (4) Our measuring setup is small, light, fully wireless, and thus adjusted to the real use cases. (5) Various ground truths (stimulus intensity and/or self-report) are available for the extended validation approaches. (6) The dataset comes with multimodal physiological data, enabling investigations to determine which physiological modalities are the most relevant for pain recognition. We hope that the publication of the dataset and its use and evaluation by the scientific community will generate additional knowledge in the field of automated pain detection.

## Methods

To overcome the shortcomings of previous datasets, we introduce a pain dataset called PainMonit Dataset (PMD). It consists of two parts that were recorded at the University of Lübeck, Germany, and the Silesian University of Technology, Poland, using physiological sensor data such as Blood Volume Pulse (BVP), Electrocardiogram (ECG), EDA, EMG, and Respiration (Resp) in combination with two distinct types of pain. Pain is a complex phenomenon that evokes different physiological responses through different types of pain, e.g., in the case of mechanical or heat stimuli^16^. To acquire pain with its various facets, heat stimuli in an experimental context and pain following a physiotherapeutic session were recorded for the two respective parts of the PMD dataset. Moreover, to enable accurate prediction for the subjective felt pain instead of a purely objective measure, the perceived level of pain was recorded and communicated by the subjects as previous work suggests its beneficial influence on Machine Learning (ML) performance^17^. Wearable devices that record biosignals have opened up new possibilities for use in naturalistic settings. However, few studies have investigated their use for pain monitoring^18^. To investigate their potential for automated pain recognition, our dataset is solely based on time series data stemming from wearable sensor devices.

The two parts of the PMD, the experimental (heat-based) and clinical one (physiotherapeutic), are named *PainMonit Experimental Dataset (PMED)* and *PainMonit Clinical Dataset (PMCD)*, respectively, and explained in more detail in the following.

### Measuring system

Monitoring the physiological state of a human being faces different obstacles. On the one hand, recording systems should be unobtrusive and integrate seamlessly into daily life. On the other hand, the success of the subsequent analysis depends on the quality of the acquired data. This is especially the case in patient monitoring or any medical application, where devices should not further complicate therapy treatment but strengthen compliance, and sufficient accuracy is required^19^. This is also true in physiotherapy, where both the therapist and the patient need to be able to move freely. What is more, there is usually a trade-off between the number of sensors available and the obtrusiveness of the overall system. However, the miniaturisation of electronics and cost reductions over the past years have enabled the introduction of a variety of increasingly small yet precise wearable devices that promise to remedy the situation. In order to keep the recording system to a minimum, two portable devices commonly used in wearable computing, the respiBAN Professional (RB) and the Empatica E4 (E4), were chosen as a common basis for data collection. This basis applies to both the experimental and the clinical trial protocols, and any differences are highlighted in the corresponding sections. The main component of the RB (respiBAN Professional, Plux, Lisbon, Portugal) is a central unit with dimensions of 54 × 85 × 10 mm and a weight of 45 g. The device is a hub for up to four wired sensors, collecting and processing data in digital format (16 bit)^20^. Each channel can record data at a sampling rate of up to 3000 Hz. The biosignals Plux kit allows the device to be fitted with different physiological sensors, such as EDA, ECG, EMG, and many more. The central unit is placed on an elastic chest belt, which acquires a Resp signal by measuring the total displacement of the thorax caused by the respiratory cycles. The wearable E4 wristband from Empatica (Empatica Inc., Boston, USA) records biomedical data such as EDA, BVP, body temperature and data from a 3-axis accelerometer sensor at sampling rates of 4 Hz, 64 Hz, 4 Hz, and 32 Hz, respectively^21^. In addition, Inter-Beats-Interval (IBI), which provides intermittent information on the time between heartbeats with a resolution of 1/64 second, and a derived Heart Rate (HR) for 10-second intervals with a sampling frequency of 1 Hz are calculated from the BVP signal directly on the device itself. Unfortunately, Empatica does not provide detailed information about the exact algorithms for the HR computation. In this study, the E4 was placed on the wrist of the non-dominant hand. Both wearables were connected via Bluetooth to a computer station. The signals from the two devices were transmitted, synchronised, integrated, and collected with a common time stamp. Moreover, the computer station with a graphical interface displayed the recorded data in real-time to ensure the data were recorded correctly.

While this hardware setup was kept similar for both parts of the dataset, there were some differences in the selection and placement of sensors used in combination with the RB, the pain induction and acquisition of a subjective perceived pain level. In particular, different sensor configurations were introduced to acquire as much and as diverse data as possible in the laboratory setup, whereas the main objective of the clinical one was to find a configuration that is compatible with physiotherapy. To reduce discrepancies as much as possible, the Resp and EDA signals were included as common modalities sharing the exact same setup between the two datasets. The Resp signal was measured using the chest belt of the RB, and the EDA sensors were attached to the *medial phalanx* of the index and middle fingers of the non-dominant hand. Regarding the differences between the datasets, an ECG sensor was applied in the setup of the PMED, whereas a second BVP at the tip of a finger was chosen as another sensor of the RB in the PMCD. Moreover, two different placements of the EMG sensor were recorded. The PMED measured the muscle activity between the shoulder and the neck (superior fibers of the *trapezius* muscle) on the side of the non-dominant arm with a reference electrode placed on the prominent spinous process of the *vertebra prominens*, the seventh cervical vertebra (*C7*) of the subject. As the sensors in the shoulder area of patients would interfere with the physiotherapeutic sessions, EMG electrodes were placed on the forehead, registering signals from the *corrugator supercilii* muscle with the reference electrode on the forehead, near the hairline in the PMCD. Furthermore, we employed one additional device in the clinical experiment: the KFORCE Grip hand dynamometer (Kinvent, Montpellier, France) aimed to get involuntary hands clenching as a reaction to pain. The device recorded the data with a sampling frequency of 75 Hz^22^. The subject held the device in their dominant hand. Table 1 summarises sensors and modalities used for PMED and PMCD with their initial recording frequency and a short description.

**Table 1:** The physiological sensor modalities collected for PMED and PMCD. The table presents the used devices, modalities, sampling frequency, and the description of details and possible differences between setups.

Device	Modality	Frequency (in Hz)	PMED	PMCD	Description/Differences
RB	ECG	500	*✓*	✗	lead III (left arm to the left foot; the reference electrode near the right arm, Einthoven configurations)
	EDA	500	*✓*	*✓*	skin electrodes on medial phalanx of the first and middle fingers, non-dominant hand
	EMG	500	*✓*	*✓*	PMED: *trapezius* muscle; PMCD: *corrugator supercilii* muscle
	Resp	500	*✓*	*✓*	resistant chest strap
	BVP	500	✗	*✓*	photoplethysmograph; clip on the ring finger
E4	BVP	64	*✓*	*✓*	photoplethysmograph; armband on the outer forearm
	EDA	4	*✓*	*✓*	dry silver plated copper backed brass electrodes on the inside of the forearm
	TEMP	4	*✓*	*✓*	infrared thermometer; forearm
	IBI	—	*✓*	✗	determined from BVP
	HR	1	*✓*	✗	determined from IBI
KFORCE	GRIP	75	✗	*✓*	held in dominant hand

**Fig. 1:**
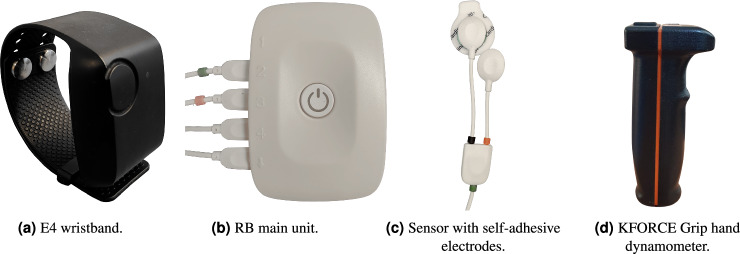
The wearable devices recording several physiological modalities during data acquisition of the PMD: (**a**) E4 wristband, (**b**) RB main unit, and (**c**) Electrodes used for the RB, i.e., EMG. The (**d**) device was used in PMCD only. Modified from Luebke *et al*.^36^, used under CC-BY 4.0 license.

All sensor modalities involving electrodes were set up using wet self-adhesive electrodes (Kendall Covidien H124SG ø 24 mm, Wolfram Droh GmbH, Mainz, Germany). Figure 1 shows the used wearable setup, namely the E4, RB, and electrodes of the RB. The details and characteristics of each part of the dataset, as well as the differences in sensor setup and pain induction and measurement between the two parts are described below.

### Experimental dataset

**Fig. 2:**
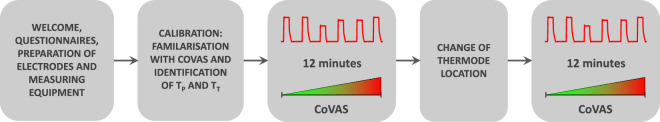
The workflow of the experimental pain study.

Performed at the Institute of Medical Informatics, University of Lübeck, Germany, the study design to collect heat pain data involved several steps. The complete workflow of the experimental protocol used to collect the PMED data is shown in Fig. 2.

For the experimental part of the dataset, 55 healthy subjects (22 male and 33 female, aged between 18 and 65 with an average of 27.47 ± 6.90) were recruited. Exclusion criteria were chronic or acute pain, pregnancy, and neurological or psychological diseases. Regular intake of medications (except contraceptives) or skin diseases in the area of thermode placement also led to exclusion. Because of technical issues during the data acquisition mainly introduced by lost Bluetooth connections or dropped electrodes, three subjects had to be removed from the final PMED. The study was conducted between August 2020 and January 2021 according to the guidelines of the Declaration of Helsinki and approved by the Ethics Committee of the University of Lübeck, Germany (19-262; protocol code 1.1, 20 September 2019). None of the participants were harmed during the experiment.

All experiments were supervised by a doctor and a physiotherapist in a quiet laboratory room, where windows were darkened, and communication with participants was standardised with regards to the protocol description and instructions provided to them. Participants were asked to wash their hands, as this directly affects the skin conductance measured on the fingers^23^, and the electrode sites were cleaned with alcoholic alcohol. The data collection was preceded by a welcome, explanation of the study and completion of informed consent and pain-related questionnaires. Moreover, participants were informed of the possibility of stopping the study at any time for any reason. The protocol also included the preparation of the electrodes and wearables, and the familiarisation of the participants with the setup.

Afterwards, the two-part data acquisition was carried out. During a *calibration phase*, individual pain thresholds were determined for each person to establish person-specific thermal stimuli, which were later induced during a *pain induction phase*.

During the calibration phase, a staircase method with steadily increasing temperature stimuli was performed to identify a pain threshold (*T*_*P*_) when heat becomes painful for the first time, and a pain tolerance threshold (*T*_*T*_) when pain becomes unbearable for subjects individually. Computerised Visual Analogue Scale (CoVAS) ratings during 10-second heat stimuli starting with a temperature of 40°C rising by 1°C up to a maximum of 49°C and intermediate pause of 5 seconds were analysed to identify the sought thresholds. Temperatures with the first CoVAS rating above 0 and 90 were marked as initial values for *T*_*P*_ and *T*_*T*_, respectively. By performing the staircase calibration method twice and averaging the found values, further robustness was introduced to the detection step. Moreover, the estimated temperature parameters were once again applied to benchmark their validity. If *T*_*P*_ was perceived too painful (CoVAS rating > 0) or *T*_*T*_ did not invoke severe pain (CoVAS rating < 90), the former was reduced by 1°C whereas the later was increased by 1°C. Eventually, *T*_*P*_ and *T*_*T*_ specified the boundaries of four painful temperature stimuli *P*_*i*_ as^7^

1 $${P}_{i}={T}_{P}+(i\times R)$$

with *i* ∈ {1, 2, 3, 4} and *R* = (*T*_*T*_ − *T*_*P*_)/4 and a non-painful temperature *NP* as

2 $$NP={T}_{P}-R.$$

**Fig. 3:**
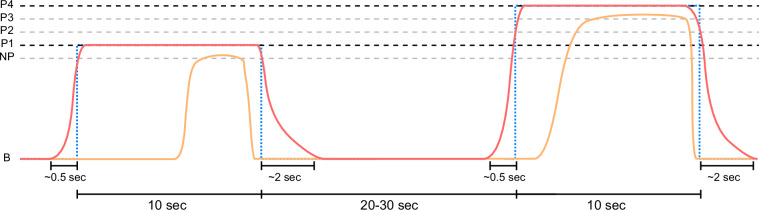
10 seconds stimuli (blue dotted lines) of the PMED with the given temperature stimulus (red curve) and possible CoVAS rating (orange curve). Modified from Gouverneur *et al*.^7^, used under CC-BY 4.0 license.

The calibration phase was followed by a pain induction phase in which participants were asked to continuously rate their subjective level of pain using the CoVAS. Each of the previously calibrated temperatures (*P*_*i*_ and *NP*) was applied eight times for 10 seconds each. The stimuli were applied in a randomised order, with rest periods of random length between 20 and 30 seconds in between, during which the temperature returned to the baseline of 32°C (*B*). With a very rapid heating rate of up to 70°C per second and a cooling rate of up to 40°C per second, the changes of the applied temperatures were performed fast by the Contact Heat-Evoked Potential Stimulator (CHEPS). An example of stimuli and CoVAS ratings can be seen in Fig. 3. The application of heat stimuli was divided into two parts with individual thermode positioning to avoid any damage from repeated heat stimuli at the same site and to reduce sensitisation or habituation effects. The exact placement of the thermode was determined by first finding the centre of the forearm, more precisely the centre between *caput ulnae* and *olecranon*, and placing the thermode 3 cm proximal and distal for the two phases. The initial placement, i.e. proximal or distal to the mid, was also randomised.

**Fig. 4:**
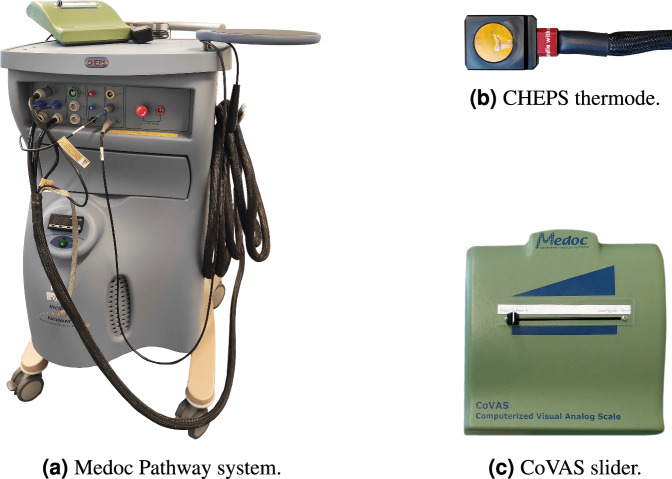
The hardware used to invoke pain and record subjective pain ratings during data acquisition of the PMED: (**a**) Medoc Pathway system. (**b**) CHEPS thermode. (**c**) CoVAS slider. From Gouverneur *et al*.^7^, used under CC-BY 4.0 license.

To enable controlled data acquisition, pain was induced by applying thermal stimuli using a Pathway CHEPS (Medoc, Ramat Yishai, Israel) following a strict protocol during the experimental study of the dataset. The stimulus-invoking machine mainly consists of a 27 mm diameter contact surface attached with a Velcro strap to the invited subjects’ non-dominant forearm (10 cm below the elbow). At all times, the temperature of the thermode was limited to 49°C following the safety limitations of the system^24^ in order to avoid harm, damage or prolonged reactions in the participants. A subjective pain level of participants was continuously captured during the data recording sessions with the help of a CoVAS. Similar to a VAS, it records a pain rating with anchors *no pain* and *worst imaginable pain* on a 10 cm line by digitalising a slider position between both anchors and mapping it to a value of 0 and 100 continuously. Figure 4 shows the pain induction machine, the contact thermode and CoVAS.

### Clinical dataset

**Fig. 5:**
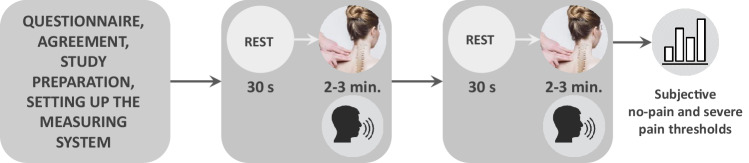
Workflow of the study performed during fascial therapy. Subjective pain level rates were given verbally during the procedure. The no pain and severe pain thresholds were acquired after the therapy was finished.

The clinical data were collected between July 2020 and finished in June 2021 at the Specialist Physiotherapy Clinic in Rybnik (Poland) and involved performing fascial therapy of the arm and neck area. Fascial therapy consists in applying pressure on the fascia, that designates the connective tissues surrounding organs, in order to relieve chronic pain^25^. It is in particular used to treat musculoskeletal pain syndromes caused by overload that result from maintaining the same body position for a long time^26^) or working in small ranges of motion, which leads to increased passive muscle stiffness and the development of pain trigger points. Fascial therapy is designed to reduce tension and improve the elasticity of the tissue. These goals are achieved by pinpoint compression or deep rubbing combined with slow movement along the muscle fibres, which improves blood circulation and thus increases tissue oxygenation^27^. The fascial layers are warmed up from superficial to deeper ones^28,29^ by gradually increasing the force transmitted by the physiotherapist’s hand^30^. The increasing intensity of therapy may result in the occurrence of pain sensations. Fascial therapy was performed on the patients of our study as part of their prescription for chronic arm and neck pain. The workflow of the study protocol is shown in Fig. 5.

The dataset consists of data collected from 49 subjects (28 female, 21 male) aged between 20 and 70 with an average of 42 ± 9.00, who were regular patients of the clinic. Due to technical issues (electrodes peeling off and motion artifacts), some patients’ data was excluded from the final dataset. The Bioethics Committee for Scientific Research at the Academy of Physical Education in Katowice (Poland) approved the study (decision number 1/2019). None of the participants were harmed during the experiment.

The procedure was carried out in an isolated, quiet therapy room by a qualified therapist. A technician and study supervisor were also present to provide assistance if needed. All patients signed a written consent after being informed in detail about the course of the procedure and the possibility of stopping the study at any moment. Information regarding age, sex, diseases, and corresponding chronic pain was then collected. The team disinfected the subject’s skin in the electrode-skin contact area and set up the measuring system. All therapy sessions were performed by one specialist.

**Fig. 6:**
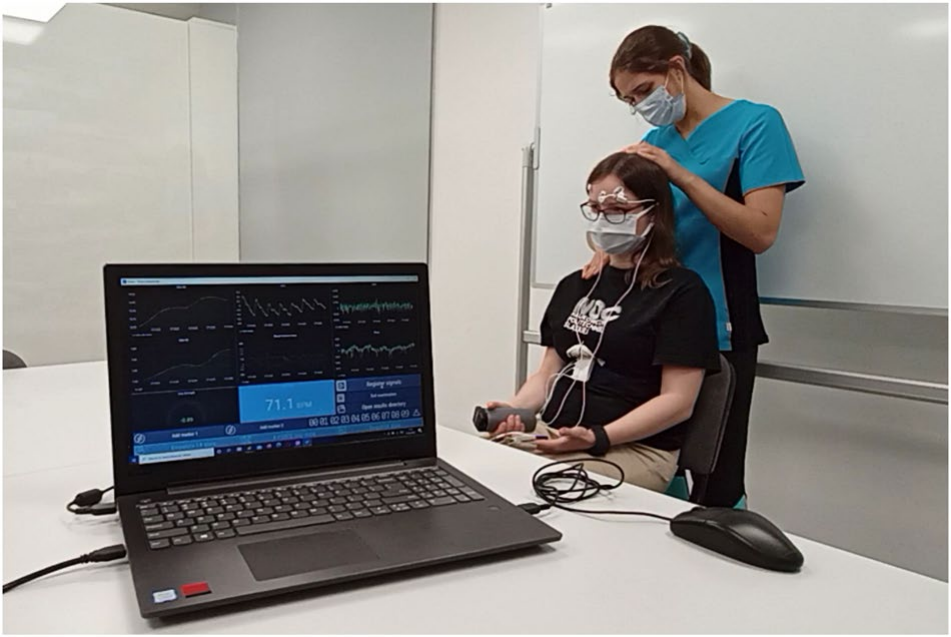
Setup of the clinical study. The wearable devices were connected to the measuring platform via Bluetooth. Acquired signals were displayed online on the computer station. To avoid additional distractions, the screen was not visible to the patient.

The fascial therapy was performed in the frame of a regular clinical visit and, therefore, could not be planned in terms of used pressure, exact time of applying stimuli, and so on. The energy transmitted through the therapist’s hands depended on the patient’s current condition and tissue sensitivity. During the study, the patient was sitting while the physiotherapist carried out the therapy standing behind him/her (Fig. 6). The measurements started with a 30-second rest period. It was followed by the 2–3-minute therapy focused on neck and arm muscles.

Since the pain stimulus in fascial therapy is not directly measurable, PMCD does not include the recording of the stimuli intensity. Moreover, as the patient should have his arms and shoulders relaxed during manual therapy, using CoVAS in such a procedure was inconvenient. Therefore, subjects were asked to rate their pain feelings with the NRS ranging from 0 to 10, where 0 stands for no pain, and 10 reflects the worst imaginable pain. Numerical values were given as frequently as possible, i.e., anytime the patient noted any changes in pain intensity and she/he was able to report it. The measuring platform allows recording pain rates in the NRS along with their corresponding timestamps by pressing numerical values in the user interface. The values communicated by the subject were inputted by the study supervisor.

The entire procedure was repeated once and was followed by a short questionnaire, where the patient indicated two numerical thresholds on the NRS: the first one below which no pain occurred and the second one above which the pain was severe. These subjective thresholds were collected after the treatment since it was impossible to do it beforehand. In particular, applying the reference force corresponding to pain at level 10 before the therapy risks damaging the tissue, which could be unprepared for such an intense stimulus. Additionally, pain sensations decrease throughout the therapy in most cases, even with similar intensity of fascial manipulation. Information on subjective pain thresholds helped to interpret and standardise the acquired data.

## Data Records

The data records of the PMD^31^ are publicly available on the following repository.

### Experimental dataset

**Fig. 7:**
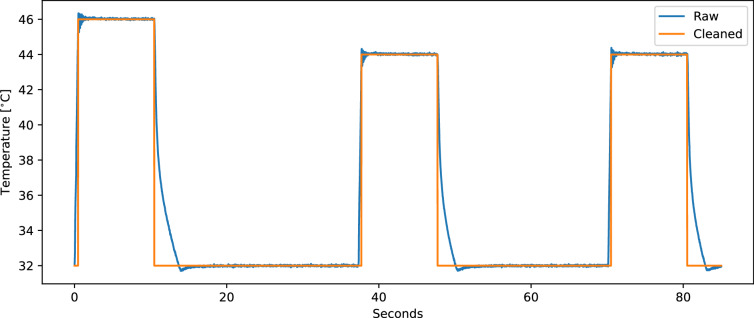
Visualisation of the measured raw thermode temperature (blue) and the processed “cleaned” signal (orange), which only represents temperatures in 0. 5°C steps, of the PMED.

The records of the PMED are made available as continuous data streams per subject in *csv* files. The *csv* files are encoded in UTF-8, where the value separator is a semicolon, and a comma represents the decimal point. The time series data for the heater thermode temperature, the CoVAS values, and the physiological sensor modalities of the RB and E4 were synchronised and resampled to a common frequency of 250 Hz. Since the intermediate pause to relocate the thermode during the pain induction phase was not fixed, the resulting data streams may vary in length across different subjects. Each stream consists of all available modalities, namely a timestamp, BVP from the E4 (BVP (E4)), EDA from the E4 (EDA (E4)), EDA from the RB (EDA (RB)), the skin temperature, IBI, HR, Resp, ECG, EMG, the thermode temperature, and the CoVAS. As the measured temperature of the thermode is subject to regular temperature variations and minor measurement errors (temperature display resolution and temperature set-point resolution are described by Medoc to be 0. 1°C), it is slightly noisy. A cleaned signal has also been generated from the raw temperature values to make it easier for researchers to analyse the data. This signal, also referred to as *Heater_cleaned*, provides temperatures in targeted 0.5-degree increments, and ensures that the stimulus windows are precisely 10 seconds long. The raw temperature signal and its resulting cleaned version are visualised in Fig. 7. Table 2 shows the first three rows of the csv file of subject 1 in a text and tabular form.

**Table 2:** Textual (upper) and tabular (lower) visualisation of the first three rows of the *csv* file from subject 1 of the PMED. For ease of presentation, figures have been rounded to two decimal places.

	

The acquired data streams can be analysed in many different ways. The training of supervised learning ML models to automate pain recognition requires data examples of fixed shapes with their associated labels. Thus, a segmentation step was applied to extract windows of physiological sensor data associated with all different pain levels. As the stimulus duration during the pain induction phase was set to 10 seconds, windows of the same size were extracted for each stimulus. In order to also retrieve examples without pain and maintain a balanced class distribution, 10-second windows were extracted precisely before the first eight stimuli during the baseline temperature. The applied temperature intensities (*B*, *NP*, and *P*_1−4_) were used to create a ground truth label for the data examples. While the baseline was encoded as 0, the non-painful stimulus and the steadily increasing painful stimuli were encoded as the number 1 and 2-5, respectively. Moreover, the labels were transformed into one-hot encoding, typically used in ML projects, for better handling of the categorical class information.

The resulting data frames were aggregated into an array and saved as *npy* files, as the *NumPy*^32^ package is one of the most commonly used libraries to handle data frames in *Python*^33^. The data and label arrays are respectively named *X.npy* and *y_heater.npy*. The data array has four axes: the number of samples, the time domain, the number of sensors and the artificially single axis introduced for ML architectures like Convolutional Neural Networks (CNNs) that expects three-dimensional input examples. For our baseline study, we decided to remove three modalities: the IBI and HR since both of them are computed by the Empatica E4 from the BVP and not raw data like the others modalities, and the skin temperature as preliminary ML studies showed a weak correlation between it and the pain annotations of our experimental dataset. Therefore, the shape of the data array is (subjects  × stimuli levels  × stimuli repetitions, seconds  × frequency, number of sensors, 1) = (52 × 6 × 8, 10 × 250, 6, 1) = (2496, 2500, 6, 1). As the first stimulus of subject 16 was unfortunately not recorded due to a technical issue, the provided dataset contains one less example (249**5**, 2500, 6, 1). The label array has a shape of (2495, 6), where its *n*-th element corresponds to the *n*-th data example in *X.npy*, and the class information is given by the one-hot encoding of the six different temperature levels. In addition to the data and label arrays, a subject information vector is also stored as *subjects.npy*, where for each data-label pair, the originating subject is encoded as an integer from 0 to 51. This enables standard evaluation techniques such as a Leave-one-subject-out (LOSO) Cross Validation (CV), which rely on testing the given model on each subject sequentially. A typical train test split of one fold in a LOSO binary classification evaluation would thus lead to train and test sizes of 816 and 16 samples, respectively. While most datasets for automated pain recognition only incorporated the stimulus intensities as ground truth for later ML models, the PMED allows for the inclusion and analysis of subjective perceived pain levels in the form of the CoVAS information. To map the CoVAS time series information to categorical classes, the sum of the ratings in one segment was computed for each segment and divided by the maximum CoVAS sum for all segments of the given subject first. Afterwards, the normalised values were binned into five categories where *C*_0_ corresponds to the value 0 and *C*_1_, *C*_2_, *C*_3_, and *C*_4_ correspond to $\left.\right]0,0.25\left.\right]$, $\left.\right]0.25,0.5\left.\right]$, $\left.\right]0.5,0.75\left.\right]$, and $\left.\right]0.75,1\left.\right]$, respectively. Again, the class labels were converted into one-hot encoding, giving a *y_covas.npy* with shape (2495, 5).

### Clinical dataset

Similarly to the PMED, therapy recordings of the PMCD are given as *csv* data streams. Since some patients agreed to participate in one session and some in two, the dataset contains one or two files per subject. Moreover, the information about the no pain and severe pain thresholds are provided in separate text files. Apart from the main therapy recording, each session goes with a corresponding rest phase (run-up) file (Fig. 5), where no stimuli were induced. Time series derived from physiological sensors were resampled to a common frequency of 250 Hz. Apart from timestamp and physiological modalities, a single *csv* file also includes *Pain rates* and *Pain labels*. *Pain rates* represent exact NRS values (0–10) given by a patient at a specific moment, whereas *Pain labels* stand for no pain, moderate pain, and severe pain classes. The labels were derived by applying individual thresholds to *Pain rates* as follows: NRS values higher than severe pain threshold were marked as severe pain (encoded with 2), values lower than no pain threshold stood for no pain (encoded with 0), and values between both thresholds represented moderate pain (encoded with 1). *Pain labels* and *Pain rates* are given with exact timestamps. Since verbal pain rates were infrequently provided by the subjects, not-rated samples were filled *NaN* values. Each stream consists of a timestamp, BVP (E4), BVP from the RB (BVP (RB)), EDA (E4), EDA (RB), the skin temperature, Resp, EMG, Grip data from KFORCE Grip hand dynamometer (KFORCE) (GRIP), *Pain rates*, and *Pain labels*. Table 3 presents a sample data stream from a single subject.

**Table 3:** Textual (upper) and tabular (lower) visualisation of three rows of the *csv* file from subject 1 of the PMCD. For ease of presentation, figures have been rounded to two decimal places.

	

**Fig. 8:**
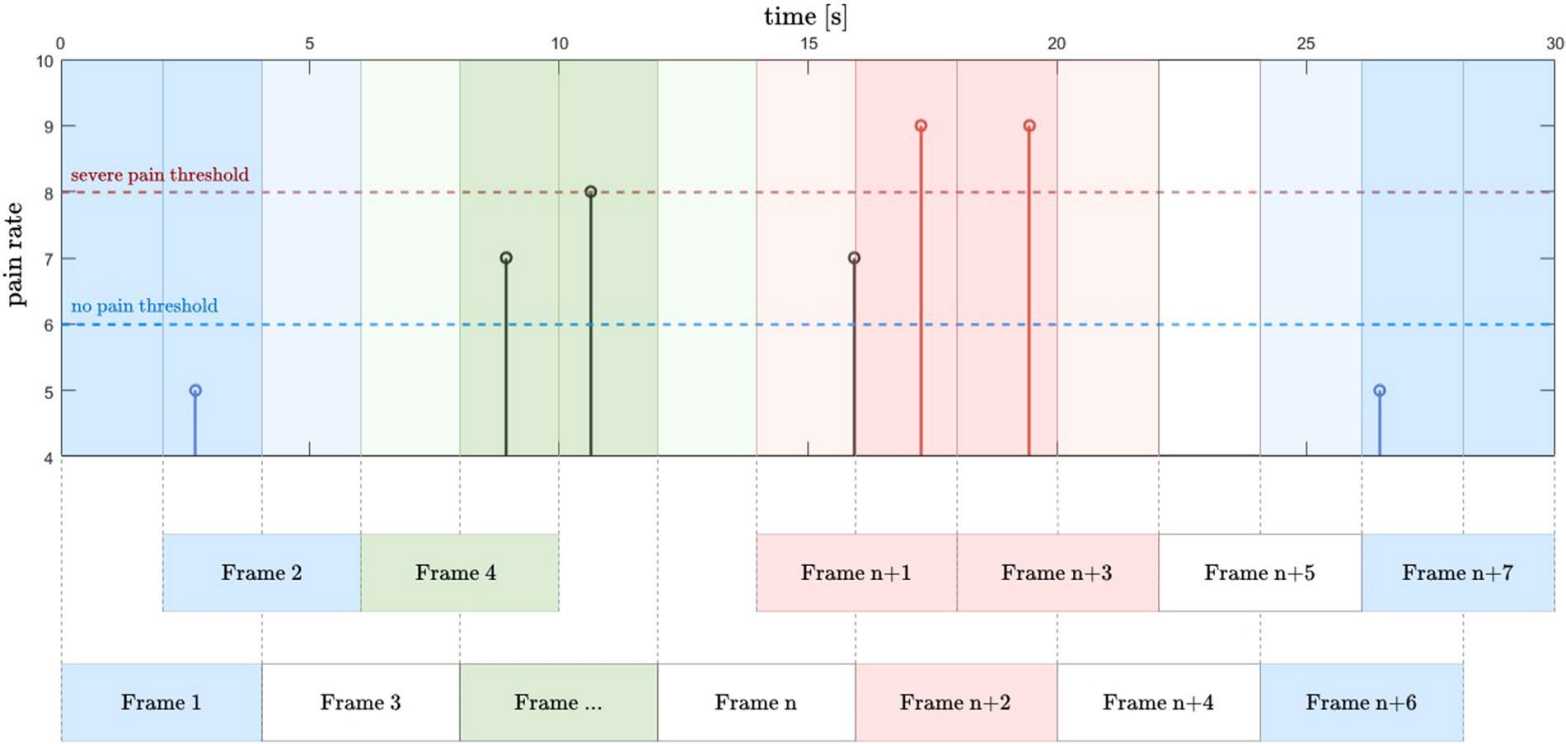
Data segmentation and frame labelling method for the PMCD. Blue, green, and red frames represent no pain, moderate, and severe pain classes, respectively. Frames 3, *n*, *n* + 4, and *n* + 5 do not hold any label and are depicted in white. Modified from Badura *et al*.^34^, used under CC-BY 4.0 license.

As pain rates are provided once every few seconds in the data streams, a segmentation of the physiological signals similar to the approach used for PMED (Fig. 8) is not optimal. Therefore, the PMCD data were segmented using a sliding window method. In our previous study^34^, we found a 4-second window with 50% overlap to be suitable for pain detection in a dynamic physiotherapy process, so such parameters were used to segment data. The resulting data frames covering any pain indication were labelled entirely with this value. For frames with more than one indication, the maximum value was used. Finally, each data frame was associated with one of the following labels: no pain, moderate pain, or severe pain, denoted by *0*, *1*, and *2*, respectively, and converted into one-hot encoding. Raw pain rates (given in NRS) are also provided. Apart from the pre-therapy frames, which all were labelled with *0*, we excluded frames that did not cover any pain indication. It is because the lack of self-report makes it impossible to assign a class (*0*, *1*, or *2*) to a frame. Sensor data includes the six physiological sensor modalities (BVP, EDA (E4), Resp, EDA (RB), ECG, and EMG). Two modalities were dropped for our baseline study: skin temperature for the same reason as for the PMED, and grip as it is not a physiological modality. The resulting *NumPy* data array *X.npy* thus has a shape of (samples, seconds  × frequency, number of sensors, 1) = (3455, 1000, 6, 1). The three classes in a one-hot encoding create a label frame (*y.npy*) of shape (3455, 3). Again, a subject vector (*subjects.npy*) encoded the association of the samples to the individual subjects. Furthermore, the class distribution for the aggregated data from all subjects is skewed: no pain and moderate pain have almost the same number of frames (1443 and 1527 respectively), whereas severe pain includes only 485 observations.

## Technical Validation

In the literature, the task of automated pain recognition is often translated into a binary classification problem, distinguishing between different levels of pain, e.g., “no pain” vs. “high pain”. Similarly, PMED and PMCD have been evaluated using ML models applied to the aforementioned *NumPy* files in several previous publications^7,34–36^. This section presents the details regarding data analysis and classification approaches for both PMD datasets.

### Experimental dataset

**Fig. 9:**
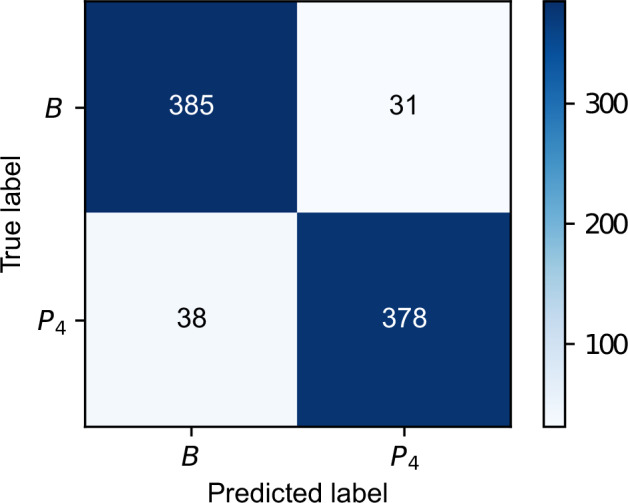
Confusion matrix for the RF approach evaluated on the PMED in a LOSO CV. An accuracy of 91.70% was obtained for the experiment. Modified from Gouverneur *et al*.^35^, used under CC-BY 4.0 license.

For PMED, automated pain recognition tools were implemented following the Pattern Recognition Chain (PRC), and several ML models benchmarked for distinguishing “no pain” (*B*) from the various pain levels (*P*_1−4_) using the physiological sensor modalities. One of the most straightforward and most transparent approaches, yet still yielding satisfying classification performance, was achieved by extracting several Hand-Crafted Features (HCFs) on the segmented samples and training Random Forest (RF)^37^ models to predict the objective heater classes. No preprocessing step was realised. Several features ranging from simple statistical ones, such as max, min, range, standard deviation, interquartile range, mean, local maxima and minima, to sophisticated modality-specific ones were retrieved for each sensor. For the EDA signal a skin conductance decomposition, analysis of the Skin Conductance Responses (SCRs) and extraction of the derivative of phasic component of EDA (dPhEDA) based on a convex EDA optimisation method (cvxEDA)^38^ and spectral features time-varying index of sympathetic activity (TVSymp), and its modified version (modified spectral features time-varying index of sympathetic activity (MTVSymp))^39,40^ was performed. Characteristics around the HR, such as the number of beats, the RR interval and the Root Mean Square of the Successive Differences (RMSSD) were extracted from the ECG and BVP. Using a trapezoidal detection^41^, the inhalation and exhalation phases in the Resp signal were estimated, and the number of breath cycles and their corresponding mean amplitudes were computed. Eventually, an aggregation of the signal power spectrum was also processed for the EMG signal. In addition to the manually extracted features, the *Neurokit 2*^42^ (neurokit2==0.2.3) *Python* library was used to retrieve all available features for the given sensor modalities. In total, a sum of 323 features was extracted from the various sensor modalities. A detailed explanation of the ML pipeline can be found in Gouverneur *et al*.^35^. Table 4 summarises the achieved classification accuracies for a LOSO CV for the various tasks and sensor modalities using the heater labels. The best accuracy of 91.93% was achieved for the EDA derived from the RB for the task *B vs*. *P*_4_. Moreover, the performance of all sensor modalities was evaluated by concatenating all available features into a common feature vector (*late fusion*) as well, yielding a slightly worse performance of 91.34%. The reported values display the maximum accuracy yielded by repeating each experiment 10 times. Figure 9 shows the confusion matrix of one “no pain” vs. “high pain” experiment, as it is one of the most commonly applied tasks in the area of automated pain recognition, indicating minor classification mistakes for the given task. Furthermore, the node impurity of the RFs allows the retrieval of feature importance for the given classification tasks. This enables further performance improvements by implementing a more sufficient feature selection process. In this study, we used Recursive Feature Elimination (RFE) which removes the least essential feature iteratively and evaluates the performance of the new feature space, yielding a best accuracy of 93.62% for the “no pain” vs. “high pain” task in a LOSO CV using 15 different features from all available sensors modalities^36^. Moreover, the present CoVAS labels allow the evaluation of the automated pain recognition system using subjective pain labels instead of objective ones, indicating further possibility to improve the classification performance^7^. The primary outcomes of the experiments underline the technical validity of the dataset by showing high classification accuracies (especially for tasks such as “no pain” vs. “high pain”).

**Table 4:** Accuracies of a RF model trained on the PMED and evaluated in a LOSO CV for several binary classification tasks.

Sensor	*B vs. NP*	*B vs*. *P*_1_	*B vs*. *P*_2_	*B vs*. *P*_3_	*B vs*. *P*_4_
Bvp	52.76	53.97	52.00	53.73	55.70
Ecg	51.92	50.60	53.57	56.13	63.57
Eda_E4	57.09	57.81	62.56	67.43	73.48
Eda_RB	55.05	64.30	67.87	79.33	91.93
Emg	52.88	52.16	50.77	51.08	52.76
Resp	50.24	50.00	55.83	52.88	56.98
All	56.85	64.06	68.45	79.81	91.34

### Clinical dataset

Four different classification tasks were defined on the PMCD: the multi-class evaluation involving all three classes (*0* vs. *1* vs. *2*), no pain vs. pain (*0* vs. *1*&*2* merged), no pain vs. moderate pain (*0* vs. *1*), and no pain vs. severe pain (*0* vs. *2*).

Four-second windows were extracted using a sliding window approach with a two-second offset for both the rest period and therapy session. Samples from the rest period were assigned the label of class *0* (no pain). Samples from the therapy session were used to constitute the pain classes *1* and *2*. More specifically, ratings on the NRS below the severe pain threshold were assigned to the class *1* and assessments above the threshold were associated with class *2*. No additional pre-processing step was applied. Feature extraction was performed in the same way as for the PMED, as described in Section *Experimental dataset*. Similarly, an RF model was used for the classification task and experiments were performed in a LOSO CV setup. Table 5 presents the obtained classification accuracies in the LOSO CV setup. The best result of 81.00% was achieved in the *0* vs. *2* experiment for the EMG modality. The *late fusion* of all modalities features resulted in an accuracy of 79.81%.

**Table 5:** Accuracies of a RF model trained on the PMCD and evaluated in a LOSO CV for several classification tasks.

Sensor	*0 vs. 1 vs. 2*	*0 vs. 1&2*	*0 vs. 1*	*0 vs. 2*
BVP	50.72	60.59	59.25	74.54
BVP_RB	49.71	60.80	57.89	74.99
EDA_E4	43.99	56.19	52.97	72.25
EDA_RB	49.48	58.04	57.07	75.92
EMG	46.71	54.49	51.71	81.00
RESP	48.87	57.82	57.37	74.74
All	55.95	64.45	62.58	79.81

To boost the classification performance, PMCD data may require some processing before segmenting data into short-term frames, as suggested by the findings of Badura *et al*.^34^. Results shown in Table 5 are based on raw data analysis solely. Therefore, we encourage dataset users to test various preprocessing solutions. Moreover, this paper presents an approach where direct numerical pain level indications are used as the ground truth. On the other hand, the pain rates can also be considered as weak labels: 1) Since maximum pain feeling could not be given before the procedure to the subject, NRS values may not be treated directly, but rather in a fuzzy manner. 2) Due to the time-discrete format of pain rates, lots of data remain unlabelled. Attempts to model missing values may boost the classification or regression results. Unsupervised or semi-supervised learning can also achieve better performance than conventional methods applied to the clinical data^15^.

## Usage Notes

To validate the PMD for automated pain recognition using ML tools, researchers can evaluate the already segmented dataset and compare it to previously published performance results^7,34–36^. The *PythonNumPy* package can be used to load the data. While the files were generated and tested using the latest versions available during the writing of the manuscript (*Python 3.12*, *pandas 2.1.1*, and *NumPy 1.26.1*), future versions should also be able to load the dataset (using the *np.load* command) due to the stable packages. The code to create the segmented dataset from the raw data streams and functions to read in the files in *Python* are shared in a *GitHub* repository with instructions on how to use it. In addition, a complete code pipeline to train RFs for the evaluation of automated pain recognition was published previously^35^. Moreover, researchers are welcome to use the raw data streams to implement their segmentation procedures, investigate regression tasks, or even analyse the data beyond the classical ML pipeline. In *Python*, the *csv* files can be easily imported using the *pandas*^43^ with the command *pd.read_csv(filename, sep”;”, decimal=”,”)*, where *filename* is the name of the file to be read in.

We believe that the presented dataset would help to address the current challenges in automatic pain recognition^15^, such as developing solutions for clinical applications. We encourage researchers to use the PMD datasets in cross-data studies. While clinical data mainly handles weak labels, the well-annotated experimental dataset may provide significant support for pain recognition systems intended for real use cases. Both PMED and PMCD include data collected with the same devices, thus the possible transfer learning attempts do not require an advanced domain adaptation process.

### Limitations

As with any study, the PMD dataset has some limitations. It can first be noted that none of the PMED or PMCD experimental protocols involved subjects reporting on their own perception of pain that could lead to some bias in self-reporting of pain, for instance via pain catastrophising^44^. To minimise the impact of potential subjective bias, standardisation to project all subjects’ ratings in the same value range could be applied as a pre-processing operation on the subjective pain ratings^7^. Further limitations are specific to each part of the dataset, and are summarised below for the experimental (PMED) and clinical (PMCD) parts separately.

As previously described, the PMED contains an artificial source of pain in the form of heat. Although this induction method can be controlled precisely, it differs from naturally occurring pain, which could limit the generalisation capacity of the trained ML models to scenarios involving the recognition of pain that elicit similar physiological responses to those of the heat stimuli. The addition of other stimuli, such as electrical, could increase the representation of different types of pain in the dataset. Moreover, the pain induction sites were limited to the subjects’ non-dominant forearm. The dataset could be enriched by including additional stimulation sites to check if the obtained performances of pain recognition can be reproduced for other pain locations.

In the case of the PMCD, the lack of information about stimuli intensity makes data segmentation challenging since the annotation of the pain and no pain periods relies on discrete self-report labels. This leads to a configuration where the data can only be weakly-labelled due to the label sparsity. Such a configuration however occurs in most clinical studies^45,46^, and provides an opportunity to develop methods for the handling of weakly-labelled data in pain assessment systems. Finally, the PMCD contains data of patients with pain limited to the arm and neck area. An interesting direction for future research may be recruiting patients experiencing chronic pain of varying origins.

## Data Availability

The PMD dataset is fully accessible to the public on the following repository. Although the dataset is publicly available, it is accompanied by a data use agreement which restricts its use to academic research.

## Code availability

The *Python* scripts for the segmentation of both the PMED and the PMCD, the generation of the cleaned temperature signal, and the import of the *NumPy* files can be found under https://github.com/gouverneurp/PMD (created on June 27, 2024). In addition, the code of all ML experiments and models evaluated on both the PMCD and the PMED (referred to as PMDB in our previous publications^7,35,36^) that were reported in Gouverneur *et al*.^35^ is publicly available at https://github.com/gouverneurp/XAIinPainResearch (created on January 27, 2023).
